# The internal aqueous phase gelation improves the viability of probiotic cells in a double water/oil/water emulsion system

**DOI:** 10.1002/fsn3.3532

**Published:** 2023-07-02

**Authors:** Shahrokh Abbasi, Alireza Rafati, Seyed Mohammad Hashem Hosseini, Shahin Roohinejad, Seyedeh‐Sara Hashemi, Hadi Hashemi Gahruie, Ali Rashidinejad

**Affiliations:** ^1^ Food Science and Technology Department Islamic Azad University Sarvestan Iran; ^2^ Department of Food Science and Technology, School of Agriculture Shiraz University Shiraz Iran; ^3^ Burn and Wound Healing Research Center Shiraz University of Medical Sciences Shiraz Iran; ^4^ Riddet Institute Massey University Palmerston North New Zealand

**Keywords:** cell viability, double emulsions, internal aqueous phase gelation, *L. plantarum*, probiotic encapsulation, Tragacanth gum

## Abstract

This research studied the viability of probiotic bacterium *Lactobacillus plantarum* (*L. plantarum)* encapsulated in the internal aqueous phase (*W*_1_) of a water‐in‐oil‐in‐water (*W*_1_/*O*/*W*_2_) emulsion system, with the help of gelation and different gelling agents. Additionally, the physicochemical, rheological, and microstructural properties of the fabricated emulsion systems were assessed over time under the effect of *W*_1_ gelation. The average droplet size and zeta potential of the control system and the systems fabricated using gelatin, alginate, tragacanth gum, and carrageenan were 14.7, 12.0, 5.1, 6.4, and 7.3 μm and − 21.1, −34.1, −46.2, −38.3, and −34.7 mV, respectively. The results showed a significant increase in the physical stability of the system and encapsulation efficiency of *L. plantarum* after the *W*_1_ gelation. The internal phase gelation significantly increased the viability of bacteria against heat and acidic pH, with tragacanth gum being the best gelling agent for increasing the viability of *L. plantarum* (28.05% and 16.74%, respectively). Apparent viscosity and rheological properties of emulsions were significantly increased after the *W*_1_ gelation, particularly in those jellified with alginate. Overall, *L. plantarum* encapsulation in *W*_1_/*O*/*W*_2_ emulsion, followed by the *W*_1_ gelation using tragacanth gum as the gelling agent, could increase both stability and viability of this probiotic bacteria.

## Graphical abstract

The stability of *W*_1_/*O*/*W*_2_ emulsion significantly increased after *W*_1_ gelation. A significant increase was observed in the encapsulation efficiency after *W*_1_ gelation. The apparent viscosity of emulsions was significantly increased after *W*_1_ gelation. *W*_1_/*O*/*W*_2_ emulsion containing gum tragacanth had the highest probiotics viability.

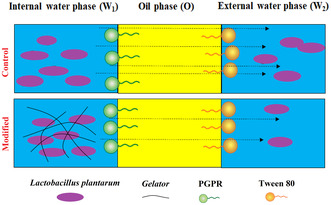

## 1 INTRODUCTION

Probiotics are live microorganisms that, when consumed at appropriate doses, possess health‐promoting effects in both humans and animals (Galdeano et al., 2019; Zendeboodi et al., 2020). Nevertheless, in the foods with health claims from the probiotics, the Food and Agriculture Organization (FAO) and World Health Organization (WHO) recommended a minimum amount of 10^6^–10^7^ CFU/g probiotic bacteria (Tanganurat, 2020).

Some important parameters can affect the survival of lactobacilli bacteria. These include the harsh conditions of the gastrointestinal tract, heat processing conditions of food (e.g., sterilization, pasteurization), and the environmental conditions (Boricha et al., 2019; Zeashan et al., 2020). In this regard, encapsulation is a well‐known approach for improving the stability of probiotics, which protects bacteria against harsh conditions and increases their viability. The potential of several encapsulation methods including emulsion, drying methods, and coacervation to increase the viability of the probiotics against environmental stresses (e.g., acidic pH, bile salt, thermal treatment, and storage period) has already been reported (Amirsadeghi et al., 2021; Dawood et al., 2019; Hashemi et al., 2020; Hosseinialhashemi et al., 2021; Lashkari et al., 2020; Mbye et al., 2020; Misra et al., 2021).

One of the most popular yet efficient systems for the encapsulation and delivery of probiotic bacteria is emulsification. Nonetheless, because of the low protective effect and stability of the conventional emulsions, double emulsions (DEs) have been evaluated as a suitable system for the encapsulation of probiotics (Muschiolik & Dickinson, 2017). Water‐in‐oil‐in‐water (*W*_1_/*O*/*W*_2_) emulsions are the emulsion systems containing dispersed oil droplets (*O*) in which tiny water droplets (*W*_1_) are dispersed and can be regarded as an emulsion in another emulsion. *W*_1_ and *W*_2_ are known as internal and external aqueous (water) phases, respectively (Xiao et al., 2017). Several studies have evaluated the feasibility of using *W*_1_/*O*/*W*_2_ emulsions for the encapsulation and delivery of probiotics. For instance, Pimentel‐González et al. (2009) reported an increase in the viability of *Lactobacillus rhamnosus* after entrapment in *W*_1_/*O*/*W*_2_ formulated with sweet whey. Pandey et al. (2021) also studied the encapsulation of LAB by *W*_1_/*O*/*W*_2_. Rodríguez‐Huezo et al. (2014) evaluated the viability of *Lactobacillus plantarum* (*L. plantarum*) loaded into the *W*_1_/*O*/*W*_2_ during the manufacture, melting, and digestion of Oaxaca cheese. Çabuk and Harsa (2012) encapsulated *L. acidophilus* in soy milk based‐DEs. In another study, the application of *W*_1_/*O*/*W*_2_ on *Bifidobacterium lactis* encapsulation for improving its viability using different wall materials was evaluated by Frakolaki et al. (2020).

The kinetic stability of *W*_1_/*O*/*W*_2_ emulsions is the main challenge in their application (Sapei et al., 2012). Several techniques and ingredients such as changes in emulsifier types (Akhtar & Dickinson, 2001), fat crystals addition, and protein−polysaccharide complexes (Sapei et al., 2012) have been utilized for improving the physical stability of DEs. There are some reports on increasing the physical stability of these systems through the gelation of *W*_1_ using some carbohydrates and proteins, and the formation of gel‐in‐oil‐in‐water (*G/O/W*) DEs (Benichou et al., 2004; Dickinson, 2011; Koubaa et al., 2018). Along these lines, the application of some proteins including sodium caseinate, whey protein, gelatin (Hemar et al., 2010), and carbohydrates such as xanthan gum (Dickinson & Lorient, 2007) has been previously reported. Moreover, the effect of polyglycerol polyricinoleate content in the oil phase (as the emulsifier and thickener) and in *W*_2_ on the final stability of DE systems was previously evaluated (Dickinson, 2011; Sapei et al., 2012). Nevertheless, as far as we are concerned, there is no research on the effects of internal water phase gelation on the viability and stability of any probiotic species in a double emulsion system. Thus, this research aimed to study the effect of *W*_1_ gelation using various gelling agents (including gelatin, alginate, tragacanth gum, and carrageenan) on the viability of L. *plantarum*, as well as various properties (e.g., size, zeta, stability, probiotic viability, entrapment efficiency, pH, rheology, and microstructure) of the *W*_1_/*O*/*W*_2_ emulsion containing this probiotic bacterium.

## 2 MATERIALS AND METHODS

### 2.1 Materials

Tween 80, gelatin, sodium alginate, carrageenan, MRS broth, and agar were purchased from Merck Co. (Darmstadt, Germany). Polyglycerol polyricinoleate (PGPR) was purchased from Danisco Co. (Copenhagen, Denmark). Tragacanth gum was obtained from Dena Emulsion Co. (Shiraz, Iran). Olive oil was supplied by Oila Company (Tehran, Iran).

### 2.2 Microorganism preparation

In the first step, one colony of *L. plantarum* (supplied by Pardis Roshd Mehrgan Co. Shiraz, Iran) was inoculated into the MRS broth and kept at 37°C overnight in a shaker incubator. The inoculum (10 mL) was then added to 90‐mL MRS broth and kept at 37°C overnight. The bacterial suspension was centrifuged at 6000*g* for 4 min at 6°C. The pellet was washed three times with sterile saline solution and then utilized in the encapsulation process.

### 2.3 Preparation of double emulsions

The *W*_1_/*O*/*W*_2_ emulsion was fabricated by a two‐step technique introduced by Bou et al. (2014). The *W*_1_ was produced by the addition of 11 log CFU/g *L. plantarum* to a sterile saline solution. Gelatin, alginate, tragacanth gum, and carrageenan were also added at 2 wt.% each in separate samples. Alginate and carrageenan samples contained CaCl_2_ (100 mM) and KCl (100 mM), respectively. After preparation, *W*_1_ was protected from light by storing it in amber tubes. Tween 80 (4% w/v) in sterile double distilled water was prepared as the external water phase (*W*_2_). PGPR (6%) was solved in olive oil and stirred at 65°C for 20 min as an oil phase (O). The *W*_1_/*O* primary emulsion was produced by adding *W*_1_ (20 g) to the *O* (80 g) phase and stirring at 500 rpm and 35°C for 20 min and homogenization at 13,000 rpm for 3 min and then 15,000 rpm for another 3 min using an IKA T25 Ultra‐Turrax (Germany). The *W*_1_/*O*/*W*_2_ emulsion was fabricated at 35°C via homogenization of *W*_1_/*O* primary emulsion (40 g) in the *W*_2_ (60 g) at 13,000 rpm for 4 min and 10,000 rpm for another 4 min.

### 2.4 Emulsion properties

#### 2.4.1 Droplet size and zeta potential

The average volume‐weighted size (D_4,3_) of samples was determined by Mastersizer 2000 static light scattering (SLS) (Malvern, UK) based on the method described by Gahruie et al. (2020). To reduce the multiple scattering effects, the samples were diluted 100 times with *W*_*2*_. The span values (indicating distribution width) were also recorded.

For the evaluation of zeta potential, the electrophoretic mobility values of diluted *W*_1_/*O*/*W*_2_ emulsions (100 times with *W*_2_) were measured by SZ100 Horiba Zetasizer (Japan) (Gahruie et al., 2020).

#### 2.4.2 Physical stability

For evaluating the stability of DEs, samples were centrifuged at 5500 *g* for 10 min. The serum volume was determined, and stability was measured by Equation (1):

(1) $$\text{Physical Stability}\;\left(\%\right)=\left(\left(V_{\left(W1/O/W2\right)}-V_{\text{serum}}\right)/V_{\left(W1/O/W2\right)}\right)\times 100$$

where *V*_(W1/O/W2)_ and *V*_serum_ are the initial volumes of *W*_1_/*O*/*W*_2_ emulsions and volume of serum phase, respectively (Hosseini et al., 2019).

#### 2.4.3 Encapsulation efficiency and viability

The *W*_1_/*O*/*W*_2_ emulsions samples (10 g) were centrifuged at 260 *g* (5°C, 15 min), followed by separating the water phase. The bacterial count of DE and the separated water phase was then determined on MRS agar medium and encapsulation efficiency (EE) was calculated using Equation (2):

(2) $$\begin{array}{c} \mathrm{EE}\;\left(\%\right)=\left(\text{Bacterial count of}\;\mathrm{DE}-\text{Bacterial count of water phase}\right)/\\ \;\text{Bacterial count of}\;\mathrm{DE} \end{array}$$

The survival of the probiotic bacterium in *W*_1_/*O*/*W*_2_ emulsions was determined weekly for 2 weeks. The total count of the probiotics in the emulsion system was determined using a serial dilution technique and then culturing on an MRS agar medium at 37°C for 24–48 h (Zhao et al., 2020). To release the encapsulated bacteria, the emulsion samples were destabilized by dilution (10^8^ times) in a sterile saline solution. The count of probiotics was reported in Log CFU/g.

#### 2.4.4 Effect of heat and pH

The encapsulated and free samples (at neutral pH) were heated for 2 min at 20, 30, 50, 63, and 72°C and then cooled to 20°C. The count of samples was determined by the agar method (Section 2.2) (Soltani Lak et al., 2021).

The influence of different pH values (i.e., 2, 3, and 6.5) on the bacteria viability for both encapsulated and free samples was determined based on the method of Sabikhi et al. (2010). pH was set by 0.1 N HCl and the sample was incubated for 1 h. The count of samples was determined by the MRS agar method.

#### 2.4.5 Apparent viscosity and viscoelastic properties

The steady shear rheological behavior of the *W*_1_/*O*/*W*_2_ emulsion system was determined at room temperature in the range of 1–100 s^−1^ by a rheometer (model MCR 302; Anton Paar) equipped with a cone and plate CP25‐1 geometry. The viscosity of the samples was determined at 50 s^−1^. The dynamic rheological properties of the samples were analyzed at 20°C by the same instrument. Amplitude (strain) sweep tests were done to evaluate the linear viscoelastic region (LVR) at the range of 0.01%–100% strain and 1 Hz. Frequency sweep test was done at the range of 0.01–10 Hz and strain of 0.01%. A 2 mL of the sample (i.e., *W*_1_/*O*/*W*_2_) was placed on the rheometer plate and allowed for equilibration and structure recovery for 5 min before the test.

#### 2.4.6 Microstructure

An optical microscope (100× magnification, model CX40; Olympus) was used for studying the microstructure of the *W*_1_/*O*/*W*_2_ emulsion. The sample was placed on the lam and covered with a lamel and then pictures were taken from different parts of the sample.

### 2.5 Statistical analysis

All tests were performed at least three times using a completely randomized design. The findings were evaluated using a one‐way analysis of variance at a .05 significance level (*p* < .05). Duncan's multiple range tests (SAS® software, ver. 9.1, SAS Institute Inc.) were applied to compare the means.

## 3 RESULTS AND DISCUSSION

### 3.1 Droplet size, zeta potential, and physical stability

**FIGURE 1:**
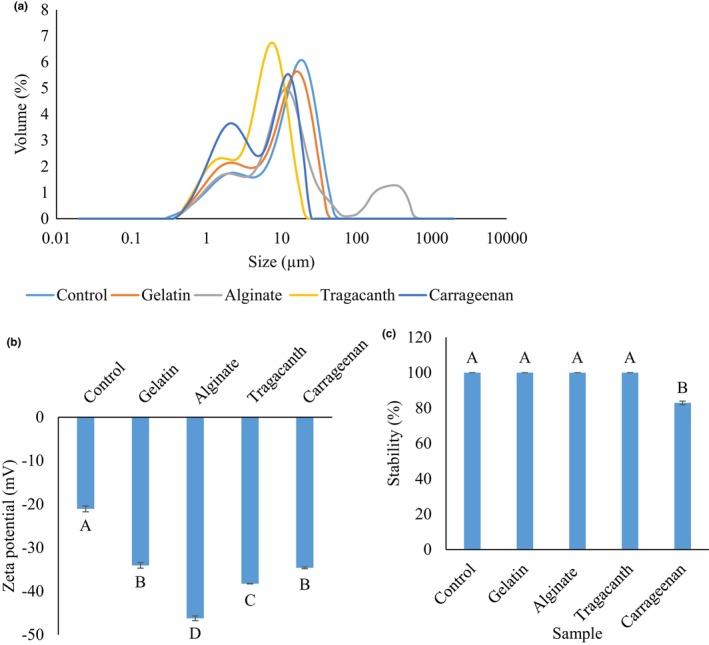
The effect of internal phase gelation using various gelling materials (i.e., gelatin, alginate, tragacanth, and carrageenan) on the (a) particle size distribution, (b) zeta potential, and (c) stability of probiotic *L. plantarum* in a double water/oil/water emulsion system. Values with different superscripted letters in each column indicate significant differences (*p* < .05).

The effect of internal aqueous phase (*W*_1_) gelation on the droplet size distribution of *L. plantarum*‐loaded *W*_1_/*O*/*W*_2_ emulsions is shown in Figure 1a. Table 1 also presents the average and span values of different samples. The droplet size of the control sample (unencapsulated), and those gelled by the addition of gelatin, alginate, tragacanth gum, and carrageenan were 14.7, 12.0, 51.1, 6.4, and 7.3 μm, respectively. The emulsion samples prepared with alginate and tragacanth gum had the largest and smallest droplet size, respectively. Application of tragacanth gum and carrageenan in the *W*_1_/*O*/*W*_2_ emulsions resulted in decreasing the size of the emulsion droplets, possibly due to reducing the coalescence of *W*_1_ droplets. This, in turn, could increase the viscous shearing forces, and reduce the re‐coalescence of *W*_1_/*O* droplets. The droplet size reduction is related to the gelled particle viscoelasticity and the resistance to coalescence after the collision between two elastic particles (Arboleya et al., 2009). The emulsions prepared with alginate had a higher droplet size, possibly due to the reason that alginate particles form large particles after interaction with calcium. Perez‐Moral et al. (2014) studied the effects of the internal phase gelation on the stability of *W*_1_/*O*/*W*_2_ emulsions and reported that the gelation with carrageenan decreased the size of water droplets by reducing the rate of re‐coalescence. The span values of control, and those gelled with gelatin, alginate, tragacanth gum, and carrageenan were 2.17, 2.32, 18.59, 1.91, and 2.77, respectively. These results show that control, gelatin, and carrageenan had the lowest polydispersity and that the uniformity of alginate and tragacanth gum was low.

**TABLE 1:** The average droplet size and span values of different samples.

Group	Average size (μm)	Span
Control	14.7	2.17
Gelatin	12.0	2.32
Alginate	51.1	18.59
Tragacanth gum	6.4	1.91
Carrageenan	7.3	2.77

Figure 1b reports the zeta‐potential values of emulsion samples. The zeta potential of the control sample, and those containing gelatin, alginate, tragacanth gum, and carrageenan in the *W*_1_ phase were −21.1, −34.1, −46.2, −38.3, and −34.6 mV, respectively. Since Tween 80 is a nonionic surfactant, the negative charge of *W*_*1*_ */O* droplets has likely resulted in forming impurities like free fatty acids present in Tween 80. The presence of anionic hydrocolloids in *W*_1_ significantly (*p* < .05) decreased the zeta potential.

Figure 1c shows the effect of *W*_1_ gelation on the stability of probiotic‐loaded emulsions after 24 h. The control sample and those containing gelatin, alginate, and tragacanth gum in *W*_1_ were completely stable after 24 h, while the *W*_1_/*O*/*W*_2_ emulsions containing carrageenan had 82.9% stability. This could be due to the release of ions from carrageenan gel to *W*_*2*_ and the change in osmotic pressure. Oppermann et al. (2015) reported that *W*_1_ gelation using gelatin and whey protein isolate increased the emulsion stability. Moreover, Perez‐Moral et al. (2014) reported that *W*_1_ gelation by alginate or carrageenan increased the stability of double emulsions by decreasing the re‐coalescence of *W*_*1*_ */O* droplets. The stability of primary *W*_*1*_ */O* emulsion has a significant effect on the stability and functionality of final DE (Dickinson & McClements, 1996). Structured *W*_1_ improves the stability of the final DE during storage (Dickinson, 2011).

### 3.2 Encapsulation efficiency

**FIGURE 2:**
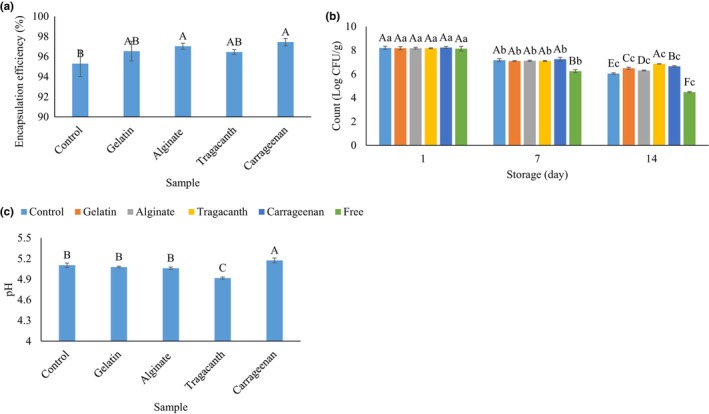
The effect of the internal phase gelation using different gelling materials (i.e., gelatin, alginate, tragacanth, and carrageenan) on the encapsulation efficiency (a) and viability (b) of probiotic *L. plantarum* in a double water/oil/water emulsion system, as well as the pH of this emulsion system (c). Values with different superscripted letters in each column indicate significant differences (*p* < .05).

Successful production of *W*_1_/*O*/*W*_2_ emulsions as a delivery system is related to their encapsulation efficiency (EE) or the amount of cargo in *W*_1_ (Schmidt et al., 2015). Figure 2a shows the effect of *W*_1_ gelation on the encapsulation efficiency of *L. plantarum*. The results of encapsulation efficiency showed that the samples containing gelation agents showed no significant differences. In the case of the current experiment, the type of hydrocolloid added to the *W*_1_ had no significant effect on the encapsulation efficiency. The encapsulation efficiency was in the range of 95.3% (the control sample) to 97.4% (in the formulation containing carrageenan in *W*_1_). This is due to the nature of DEs and the existence of different layers around the core water. DEs system as a suitable technique with high encapsulation efficiency for the encapsulation of microorganisms was introduced previously (Jiang et al., 2021; Wang et al., 2020).

### 3.3 Viability and pH

An effective encapsulation system for probiotics should improve their viability and protect them from harsh conditions during processing and gastrointestinal digestion (Beldarrain‐Iznaga et al., 2020). The effect of *W*_1_ gelation on the viability of *L. plantarum* encapsulated in *W*_1_/*O*/*W*_2_ emulsions is reported in Figure 2b. In all samples, the viability was significantly decreased during the 14 days of storage at 7°C. The highest decrease was observed in the case of the unencapsulated sample. The incorporation of the probiotic bacterium into the *W*_1_ improved its viability, confirming the potential application of *W*_1_/*O*/*W*_2_ emulsions for probiotic encapsulation. The gelation of *W*_1_ further improved the viability of *L. plantarum*. At the end of storage, the emulsion sample containing tragacanth gum in *W*_1_ had the highest viability (6.85 log CFU/g). The protection of *L. acidophilus* against the gastrointestinal condition and bile salts has been reported in previous studies after its incorporation into *W*_1_ (Shima et al., 2006). Similarly, Pimentel‐González et al. (2009) reported an improvement in *L. rhamnosus* viability after its encapsulation in *W*_1_/*O*/*W*_2_ emulsions prepared with sweet whey. Rodríguez‐Huezo et al. (2014) evaluated the viability of *L. plantarum* encapsulated in DEs during the manufacture, melting, and digestion of Oaxaca cheese. The *W*_1_ phase consisted of either sweet whey or aguamiel. Çabuk and Harsa (2012) encapsulated *L. acidophilus* in soy milk based‐DEs. Frakolaki et al. (2020) evaluated the effects of different wall materials on the viability of *Bifidobacterium lactis* encapsulated in *W*_1_/*O*/*W*_2_ emulsions. DEs were able to significantly increase the viability of this probiotic bacteria during 30 days of storage under refrigeration and freezing conditions; however, the viability in the conventional extrusion system was decreased to about 46.8% after 15 days of storage.

The effect of W_1_ gelation on the pH of *L. plantarum*‐loaded *W*_1_/*O*/*W*_2_ emulsions after preparation and storage at 7°C in the current study is reported in Figure 2c. The pH of all samples was in the close range from 4.92 to 5.17. These results showed that the pH of emulsions did not significantly affect the viability of encapsulated probiotics. Samples containing carrageenan had the highest pH value (5.17) while those with tragacanth gum had the lowest pH value (4.92). These results show that the pH of samples is in a suitable range for their application in food products.

### 3.4 Viability against heat

The effect of various heating processes on the viability of free and encapsulated *L. plantarum* is shown in Table 2. Heat processing is one of the main processes in the food industry and can reduce the viability of bacteria. The results showed that L. *plantarum* encapsulated in different types of internal phase gelation had desirable stability against various temperatures (30, 50, 63, and 72°C for 2 min). Different heat processing and the internal phase gelation showed different influences on the *L. plantarum* viability. In the case of unencapsulated *L. plantarum*, their count reduced from 9.95 log to 9.97, 8.10, 6.37, and 0 log after heating at 30, 50, 63, and 72°C, respectively. Heating at 72°C reduced the count of free bacteria to 0, while this sample did not show any viability. In the case of the control sample, the treating process significantly reduced the count from 9.92 log CFU/mL to 5.74 log CFU/mL after heating at 72°C. The *L. plantarum* count in gelatin, alginate, carrageenan, and tragacanth gum remained constant after heating at 30°C, while it reduced at 63°C and 72°C for 2 min, respectively. The tragacanth sample had the highest stability among all samples. The count of the tragacanth sample after heating at 63 and 72°C was 8.12 and 7.33 log CFU/mL, respectively. The same findings were reported by Kim et al. (2001). They reported that the viability of *L. acidophilus* after heating at 60°C for 1 h significantly reduced (above 90%). Also, in a study by Mandal et al. (2006), it was shown that the heating process at 55, 60, and 65°C for 20 min significantly decreased the count of unencapsulated bacteria.

**TABLE 2:** The effect of heating (0, 40, 60, and 80°C) on the viability of probiotic *L. plantarum* encapsulated within the internal phase of gels made using various gelling materials (i.e., gelatin, alginate, tragacanth, and carrageenan) in a double water/oil/water emulsion system.

Group/heating	20	30	50	63	72
Free bacteria	9.95 ± 0.11^Aa^	9.97 ± 0.12^Aa^	8.10 ± 0.16^Bb^	6.37 ± 0.11^Dc^	0.00 ± 0.00^Dd^
Control	9.92 ± 0.04^Aa^	9.94 ± 0.08^Aa^	8.12 ± 0.13^Bb^	7.41 ± 0.24^Cc^	5.74 ± 0.05^Cd^
Alginate	9.93 ± 0.08^Aa^	9.86 ± 0.03^Aa^	8.06 ± 0.11^Bb^	7.85 ± 0.12^Bc^	6.77 ± 0.14^Bd^
Kappa carrageenan	9.86 ± 0.03^Aa^	9.94 ± 0.09^Aa^	8.11 ± 0.18^Bb^	7.85 ± 0.05^Bc^	6.82 ± 0.07^Bd^
Gelatin	9.92 ± 0.09^Aa^	9.85 ± 0.08^Aa^	8.08 ± 0.14^Bb^	7.94 ± 0.06^ABb^	6.69 ± 0.16^Bc^
Tragacanth gum	9.98 ± 0.11^Aa^	9.87 ± 0.09^Aa^	8.49 ± 0.31^Ab^	8.12 ± 0.04^Ac^	7.33 ± 0.24^Ad^

*Note*: Data represent mean ± standard deviation of three independent repeats. Values with different superscripted letters in each column indicate significant differences (*p* < .05).

### 3.5 Viability against the acidic condition

The viability of probiotic bacteria against acidic pH is one of the main parameters in the application of this ingredient. The viability of probiotic bacteria is reduced after decreasing the pH of foods. The effects of various pH (2, 3, 6.5, and 7) on the free and encapsulated *L. plantarum* in this study are reported in Table 3. The viability of *L. plantarum* was significantly reduced at pH 2 and 3 and the highest viability was observed in the natural pH (6.5 and 7). The viability of encapsulated bacteria was higher than free bacteria and between encapsulated bacteria, the sample containing tragacanth gum had the highest viability. This could be due to the prebiotic effect of tragacanth. While tragacanth is a good coating material for various food ingredients, it contains prebiotic properties as well. Chandramouli et al. (2004) reported that the *Lactobacillus acidophilus* encapsulated in the alginate bead increased their viability at pH 2. Krasaekoopt et al. (2004) reported that the viability of *L. acidophilus* in gastric juice was increased after its encapsulation in alginate. Similarly, Afzaal et al. (2019) reported that encapsulation could increase the viability of probiotics in acidic conditions. Additionally, several other researchers reported that the encapsulation of some probiotics (*L. acidophilus* and *L. paracasei*) increased the viability in acidic conditions (pH 2 for 2 h) (Afzaal et al., 2019) and the acidic food product (pH 4.6 for 21 days) (Kia et al., 2018).

**TABLE 3:** The effect of pH (2, 3, 6.5, and 7) on the viability of probiotic *L. plantarum* encapsulated within the internal phase of gels made using various gelling materials (i.e., gelatin, alginate, tragacanth, and carrageenan) in a double water/oil/water emulsion system.

Group/pH	7	6.5	3	2
Free bacteria	9.95 ± 0.11^Aa^	9.97 ± 0.09^Aa^	5.74 ± 0.09^Eb^	5.41 ± 0.13^Ec^
Control	9.92 ± 0.04^Aa^	9.89 ± 0.08^Aa^	6.91 ± 0.04^Db^	6.75 ± 0.06^Dc^
Alginate	9.93 ± 0.08^Aa^	9.90 ± 0.05^Aa^	7.59 ± 0.08^Cb^	7.36 ± 0.12^Cc^
Kappa carrageenan	9.86 ± 0.03^Aa^	9.89 ± 0.09^Aa^	7.81 ± 0.08^Bb^	7.63 ± 0.06^Bc^
Gelatin	9.92 ± 0.09^Aa^	9.87 ± 0.07^Aa^	7.50 ± 0.04^Cb^	7.38 ± 0.11^Cb^
Tragacanth gum	9.98 ± 0.11^Aa^	9.87 ± 0.05^Aa^	8.05 ± 0.06^Ab^	7.88 ± 0.04^Ac^

*Note*: Data represent mean ± standard deviation of three independent repeats. Values with different superscripted letters in each column indicate significant differences (*p* < .05).

### 3.6 Apparent viscosity

**FIGURE 3:**
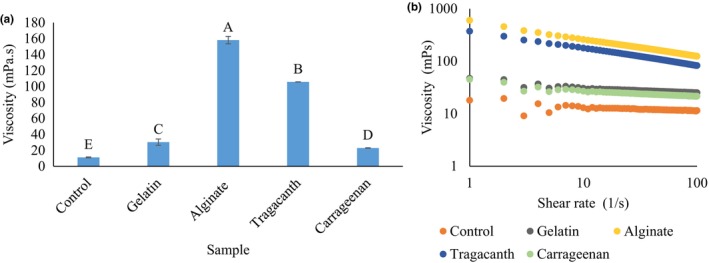
The effect of internal phase gelation using various gelling materials (gelatin, alginate, tragacanth, and carrageenan) on the viscosity at 50 s^−1^ (a) and viscosity‐shear rate dependency (b) of probiotic *L. plantarum* in a double water/oil/water emulsion system. Values with different superscripted letters in each column indicate significant differences (*p* < .05).

Figure 5a,b shows the effect of *W*_1_ gelation on the apparent viscosity and rheological behavior of *L. plantarum*‐loaded *W*_1_/*O*/*W*_2_ emulsions, respectively. The gelation of *W*_1_ significantly increased the apparent viscosity. At the shear rate of 50 s^−1^, emulsions containing alginate had the highest value of apparent viscosity (158.1 mPa s), while the lowest viscosity was observed for the control (11.2 mPa s). The variations in the rheological behavior of the *W*_1_/*O*/*W*_2_ emulsions containing different hydrocolloids might be related to the intrinsic parameters of biopolymers such as molecular weight, charge density, and conformation. There is a possibility that some hydrocolloid molecules diffuse from *W*_1_ to *W*_2_ during the final homogenization step, or storage and increase the viscosity of the continuous (external) aqueous phase (*W*_2_). Hydrocolloids can organize a large amount of water, increase viscosity, and finally improve the stability of an emulsion system (Hashemi Gahruie et al., 2020). In the current experiment, shear‐thinning (pseudoplastic) behavior was observed in *W*_1_/*O*/*W*_2_ emulsions (Figure 3). In these fluids, the viscosity was reduced by raising the rate of shear in various samples. Shear‐thinning behavior could be the result of decreasing the interaction during spindle rotation and molecular realignment of the emulsifiers and gelling agents during spindle rotation. This behavior could be attributed to the deflocculation of oil droplets. Similar findings were reported by Hashemi Gahruie et al. (2020) about the apparent viscosity of W/O/W double emulsion prepared by a similar composition.

### 3.7 Viscoelastic properties

**FIGURE 4:**
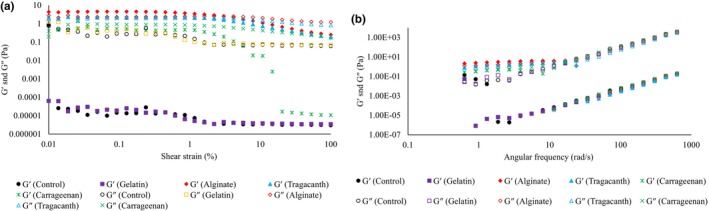
The effect of internal phase gelation using various gelling materials (gelatin, alginate, tragacanth, and carrageenan) on the viscoelastic properties of probiotic *L. plantarum* double W/O/W emulsion: (a) amplitude sweep, (b) frequency sweep test.

Emulsion systems can possess viscoelastic properties, depending on droplet concentration and particle size distribution. Different molecular and microscopic parameters can affect the rheological properties of emulsions (Deshpande, 2010). The results of the strain sweep test carried out in this study are shown in Figure 4a. Except for *W*_1_/*O*/*W*_2_ emulsion containing alginate in *W*_1_, other samples revealed dominant viscous behavior (*G*″ > *G*′) in the linear viscoelastic region. The predominant elastic character of DEs containing alginate (*G*′ > *G″*) agreed with the apparent viscosity results. Figure 4b reports the results of the frequency sweep test. The *W*_1_/*O*/*W*_2_ emulsions containing alginate in *W*_1_ had higher values of *G*′ and *G*″ than other samples. All samples exhibited a liquid‐like behavior (*G*″ *> G*′) over the studied frequency range, which means the presence of hydrocolloids in *W*_*1*_ */O* primary emulsion droplets could not affect the bulk rheological properties of final *W*_1_/*O*/*W*_2_ emulsions. The phase matrix, the oil–water interfacial properties, and oil volume fraction influence the rheological characteristics of emulsions (Dickinson, 2012; Geremias‐Andrade et al., 2016). Ye and Taylor (2009) reported that the droplet size distribution of geld emulsions was suitable and homogeneity in cold‐set emulsion gel in comparison with the conventional emulsion was higher. These results were also confirmed by Boutin et al. (2007). Lorenzo et al. (2013) evaluated the rheological properties of the acyl gellan gum stabilized emulsion gel system. They reported that the rheological properties of the internal phase gelled sample were higher than the internal phase liquid sample.

Different researchers have evaluated the effects of biopolymers including pectin (Dickinson & James, 2000), xanthan gum (Bryant & McClements, 2000), locust bean gum (Rocha et al., 2009), κ‐carrageenan (Çakır & Foegeding, 2011), and basil seed gum (Rafe et al., 2013) on the rheological characteristics of various gel systems. They reported that hydrocolloids could influence the rheological parameters of protein gels at neutral pH. This phenomenon is due to the thermodynamic incompatibility between the hydrocolloids during the formation of a gel. The addition of hydrocolloids can promote the concentration of whey proteins in the water phase and subsequently, change the process of gel formation (Çakır & Foegeding, 2011; Spotti et al., 2012). Overall, the gel properties of a protein can be improved by hydrocolloids such as proteins and carbohydrates. Turgeon and Beaulieu (2001) reported that hydrocolloids (Pectin and Carrageenan) changed the water droplet distribution between molecules and accelerated the gelation preparation at a critical concentration of gelling agent.

### 3.8 Microstructure

**FIGURE 5:**
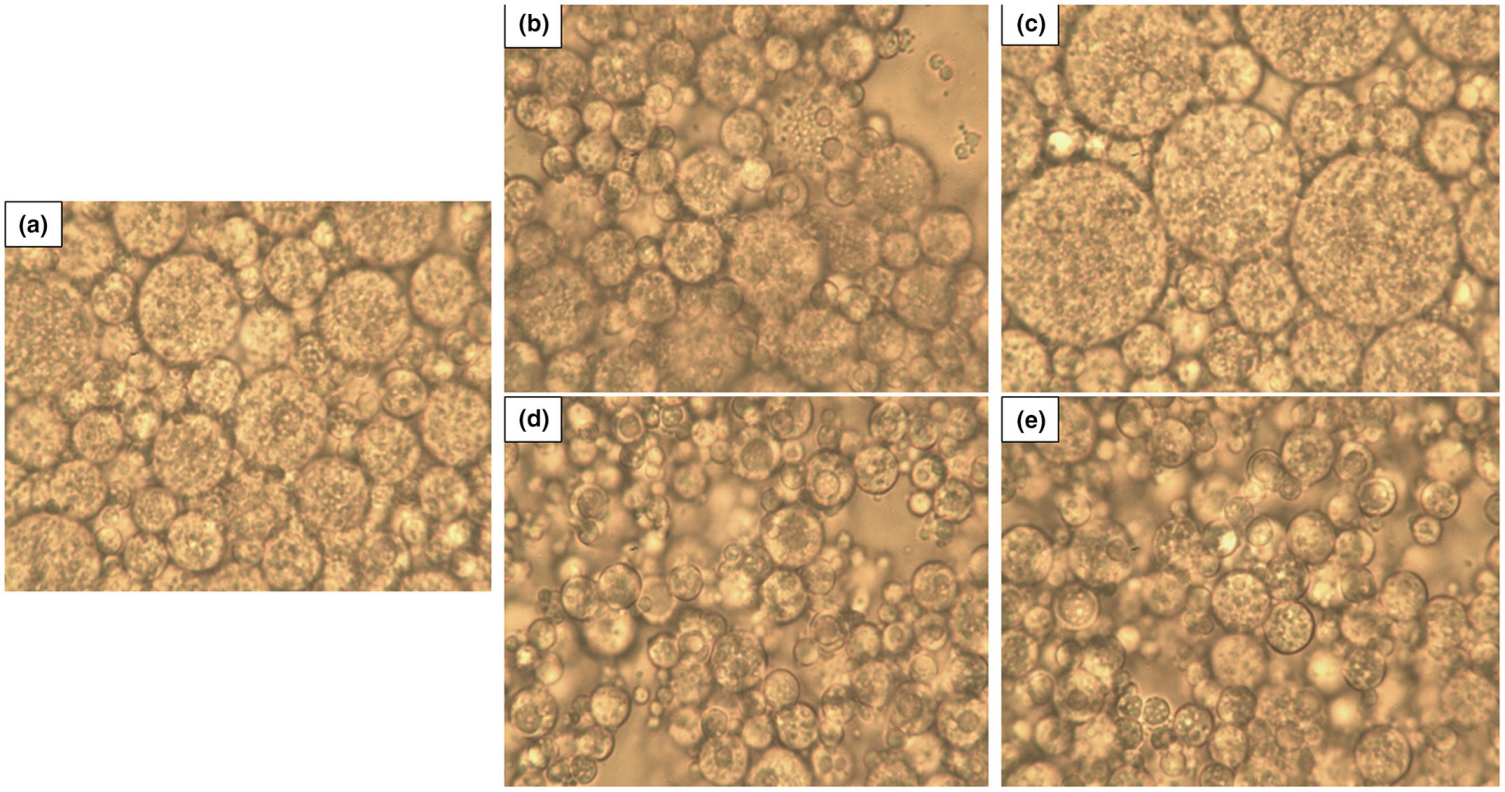
The effect of internal phase gelation using various gelling materials (i.e., gelatin (b), alginate (c), tragacanth (d), and carrageenan (e) vs. the control sample (a)) on the microstructural properties of probiotic *L. plantarum* in a double water/oil/water emulsion system.

Figure 5 shows the microstructure of control and *W*_1_/*O*/*W*_2_ emulsions containing hydrocolloids in *W*_1_. The optical micrographs confirmed the formation of *W*_1_/*O*/*W*_2_ double emulsions. These results showed that the addition of different hydrocolloids did not have any negative effect on the formation of *W*_1_/*O*/*W*_2_ emulsions. The fabrication of such emulsions using this technique has been previously reported by Hosseini et al. (2019) and Kheynoor et al. (2018). These results show that the addition of gelling agent can increase the viability of the probiotic bacteria without any negative effect on DE production. In addition, these findings demonstrate a higher internal water phase for the encapsulation of probiotic bacteria.

## 4 CONCLUSIONS

The application of *W*_1_/*O*/*W*_2_ emulsions and the internal phase gelation method for the encapsulation of *L. plantarum* allowed for obtaining a suitable delivery system for this probiotic bacterium. The use of tragacanth gum in *W*_1_/*O*/*W*_2_ emulsions resulted in increasing the encapsulation efficiency and decreasing particle size. Furthermore, the system containing encapsulated *L. plantarum* showed superior stability and rheological parameters in comparison with the control, while the stability of *L. plantarum* encapsulated in *W*_1_/*O*/*W*_2_ emulsions was improved during the 14 days of storage. Thus, a double emulsion system with internal water phase gelation can improve the viability of probiotic bacteria such as *L. plantarum* against heat and acidic conditions. In this regard, in the case of the current study, tragacanth gum was shown as the best protective agent. The survival of the encapsulated *L. plantarum* during the storage, as the result of the new delivery system developed in this experiment, demonstrates this technique as a promising alternative delivery approach for such a probiotic bacterium. The encapsulated probiotic has the potential to be incorporated into various food systems to increase probiotic viability during storage and deliver viable *L. plantarum* to the human body.

## AUTHOR CONTRIBUTIONS

**Shahrokh Abbasi:** Conceptualization (equal); investigation (equal); methodology (equal); software (equal); writing – original draft (equal). **Alireza Rafati:** Conceptualization (lead); methodology (equal); project administration (lead); resources (equal); supervision (equal); validation (equal). **Seyed Mohammad Hashem Hosseini:** Conceptualization (equal); methodology (equal); project administration (equal); writing – review and editing (equal). **Shahin Roohinejad:** Supervision (equal); validation (equal); writing – review and editing (equal). **Seyedeh‐Sara Hashemi:** Investigation (equal); methodology (equal); software (equal); writing – review and editing (equal). **Ali Rashidinejad:** Writing – review and editing (equal). **Hadi Hashemi Gahruie:** Formal analysis (equal); methodology (equal); software (equal).

## FUNDING INFORMATION

This research did not receive any specific grant from funding agencies in the public, commercial, or not‐for‐profit sectors.

## CONFLICT OF INTEREST STATEMENT

The authors declare that they have no conflict of interest.

## Data Availability

The data that support the findings of this study are available on request from the corresponding author. The data are not publicly available due to privacy or ethical restrictions.
